# Reviews and Book Notices

**Published:** 1881-03

**Authors:** 


### Reviews and Book Notices.

ARTICLE XIII.—A PRACTICAL TREATISE ON NERVOUS EXHAUSTION (NEURASTHENIA); ITS SYMPTOMS, NATURE, SEQUENCES, TREATMENT. By George M. Beard, A.M., M.D., Fellow of the N. Y. Academy of Medicine; of the N. Y. Academy of Sciences; Vice-President of the American Academy of Medicine, etc. 8vo., cloth, pp. xx 198; New York: William Wood & Co. 1880.

This is an eminently American book. The disease of which it treats is much more common in this country than in Europe, and most of its literature was published in the United States, originating with Dr. Beard himself. This fact will bring a difficulty in the way of any reviewer, for one can hardly blame an inventor for an excessive admiration of his discovery, or for extolling it in all directions.

Neurasthenia is the name under which spinal irritation (Hammond), idiosyncrasies and morbid fears, cerebral irritation, hypochondriasis, and many symptoms of an exhausted nervous system are classed. It treats of functional diseases, and is a connecting link between the disease of the body and those of the mind. It is hoped that it will open a new and important field of inquiry into the latter.

It seems that one cause of neurasthenia has been overlooked, or very little appreciated by the author; it is the proper training of the mind. Let the reader imagine an Indian, or any wild man, thrown all at once in the midst of advanced civilization, and he can form an idea of his awkwardness, and the desperate struggle of his mind to grapple with the difficulties of the situation. He may not be able to stand it at all. Every one of us is born an ignorant being. Little is our stock of inherited knowledge, though a peculiar disposition for acquiring it surely passes from parents to children. Until a comparatively advanced age, very little has been learned about the million intricacies of human life and civilization, yet if one stops learning at any period of his life, he must remain ignorant of many facts; his faculties will remain rudimentary in that direction, and he will exhibit those rude, whimsical, or eccentric peculiarities, which, after all, are nothing but a lack of the necessary training. His brain will remain weak and childish from a want of proper culture. Nay, some persons will take pleasure in encouraging these fancies; vital energy must expend itself, and many are the insane who owe it to themselves for their wretched condition. Life in a highly civilized community is a hard trade to learn, many are its drawbacks, yet it lacks its schools and its teachers; while remedies will do little for idiosyncrasies and morbid fears, despite the assertions of Dr. Beard.

This book is highly interesting, but, we are sorry to say, has too much in common with the pamphlets of advertising physicians.

Ergot, arsenic, cannabis indica, opium, caffeine, zinc, the bromides, chloral, strychnia, electricity and massage are the drugs and remedial agents relied upon by the author, with greater attention to hygiene than is recommended in most diseases.

H. D. V.

ARTICLE XIV.—TRANSACTIONS OF THE MINNESOTA STATE MEDICAL SOCIETY FOR THE YEAR 1880. St. Paul: H. M. Smyth & Co.

A copy of the proceedings of the State Medical Society of Minnesota is at hand and reflects great credit upon the profession of that progressive commonwealth.

Within its pages are discussed some very important topics. The essay upon "The Law of Heredity," by Dr. Daniel Leasure, of St. Paul, is a very well written *résumé* of the views of the best authorities upon that subject. Dr. Emery, of Minneapolis, contributes an article upon "Medical Education," in which he advocates the general use of the metric system among physicians.

The article entitled "Cardiac Hypertrophy, with Valvular Lesions," by J. P. Squires, M.D., is the report of a very interesting case. It is illustrative of the different opinions held by different practitioners concerning the same case.

The *lack of an index* is a serious defect in this volume and detracts much from its completeness as a good specimen of bookmaking. B. W. G.

ARTICLE XV.—THE MICROSCOPE AND MISCROSCOPIC TECHNOLOGY; A TEXT BOOK FOR PHYSICIANS AND STUDENTS. By Heinrich Frey, Professor of Medicine in the University of Berlin. Translated and edited by George R. Cutter, M.D., New York.

This work has passed through several German and one English edition, and is already well known to the English speaking public. This edition has been improved by the addition of new matter, a better type and paper, and the enlargement of the pages. The notes of the American editor are enclosed in brackets and add much to the value of the book. In the department of pathology we find these pages remarkably scant, the author referring to it only as morbid physiology, and the work consists, after the first few chapters devoted to the mechanisms, testing and manipulation of the microscope, and the preparation and mounting of objects, of a close exposition of microscopical anatomy. We regret to see so many of the cuts destitute of the clearness requisite to accurate illustration, and in Section IV, on "Testing the Microscope," occurs an apparent discrepancy of statement, which in some future edition we would like to see explained. The author tells us that a good objective, magnifying eighty or one hundred times, should, with the proper illumination, enable one to recognize the systems of lines sharply and distinctly on each scale of the Pleurosigma Angulatum, while weaker objectives, magnifying forty or fifty times, should show something of the lines. To the same paragraph is appended an illustration of this well-known test diatom apparently magnified at least four hundred and fifty times. Further on we read that Hortnack's newly-constructed No. 9 will resolve the lines clearly; likewise his No. 8. Then, turning to the price list of this manufacturer, in the appendix, we find the magnifying power of these systems with the lowest eye pieces stated as four hundred and ten and two hundred and fifty. There are also a number of inacuracies in the index.

These faults indicate hasty preparation and revision, ill-suited to so important an adjunct to the medical library as this work is intended to be. W. L. D.

ARTICLE XVI.—THE MICROSCOPIST. A Manual of Microscopy and Compendium of the Microscopic Sciences; Micro-mineralogy, Micro-chemistry, Biology, Histology and Practical Medicine. Fourth edition; enlarged, with 252 Illustrations. By J. H. Wyeth, M.D., Professor of Microscopy and Histology in the Medical College of the Pacific, San Francisco, California. Philadelphia: Lindsay & Blakiston.

This excellent work has been evidently very carefully prepared, and the amateur microscopist and studious physician will find in it a very satisfactory aid to study. While matters of mere curiosity receive but brief attention, every necessary fact and principle relating to microscopy is carefully stated and classified.

Practical medicine receives most attention, and the chapters on histology, pathology, diagnosis and etiology constitute the larger part of the book. They are finely illustrated; most of the cuts in the three latter being very clearly transferred from Rindfleisch. Many of the colored illustrations are fine, and an excellent thing we notice is that, appended to most of the cuts are figures showing the amount of enlargement of the subject in diameter. Owing to the progress of microscopic science and the constant improvement in optical invention it has been found necessary to provide an appendix in which microscopic technology is brought well up to date. An index and glossary of microscopic terms closes the book. It has evidently been the author's intention to furnish, not so much an exhaustive treatise as a *résumé* of microscopy which will enable the student in any department to pursue original investigations with a general knowledge of what has been accomplished by others, especially considering the needs of physicians and students of medicine. W. L. D.

Article XVII.—A Practical Treatise on Fractures and Dislocations. By Frank Hastings Hamilton, A.M., M.D., LL.D., Surgeon to Bellevue Hospital, New York; Consulting Surgeon to Hospital for Ruptured and Cripples; to St. Elizabeth Hospital, etc.; Author of a Treatise on Military Surgery and Hygiene, a Treatise on the Principles and Practice of Surgery, etc. Sixth American Edition, revised and improved. Illustrated with 352 wood cuts; 8vo, pp. 909. Philadelphia: Henry C. Lea's Son & Co. 1880.

Extract from preface to the sixth American edition:

" The present work remains, therefore, the only complete treatise on fractures and dislocations, in any language, except the treatise of Malgaigue, which latter has undergone no revision or republication since the date of its first edition " (1855).

The present work is the pride of American surgical literature. It is so well known by the profession at large that it seems useless to state that no surgeon should be without it.

The author is endowed with that soundness of judgment and criticism which few writers possess on subjects which they have made a specialty of, and this did not contribute little towards the well-deserved popularity of a book which has in twenty years reached its sixth edition.

It is handsomely printed, and beautifully illustrated with cuts, several of them new, that are indispensable in such a work, and represent all the latest improvements in mechanical surgery. It contains a chapter on General Prognosis, and one hundred pages more than the preceding edition. A large part of it has been re-written. H. D. V.

Article XVIII.—Minor Surgical Gynæcology. A Manual of Uterine Diagnosis and the Lesser Technicalities of Gynæcological Practice; For the Use of the Advanced Student and General Practitioner. By Paul T. Mundé, M.D., Professor of Gynæcology in Dartmouth Medical College, etc.

The subscribers to Wood's Medical Library will find this a very satisfactory addition to it. It is written for the class, to supply whose needs this series was first introduced, the general practitioners. While its necessity may not impress itself upon the gynæcological expert who by years of practice and research has familiarized himself with every detail of his specialty, or to the favored student who has secured to himself a practical course in gynæcology; yet to the large majority of the medical profession these practical chapters will be of great value. Emmet's maxim, "Success in the treatment of the diseases of women lies wholly in attention to minute details," sounds the key-note of these pages. Nor do historical lumber or literary research encumber them, but each one presents valuable hints for the daily use of the beginner in gynæcological art, so clearly is every digital and instrumental detail of the various manipulations and minor operations, which he is liable to meet with every day, laid before him.

The work is illustrated by three hundred wood cuts of instruments, tables, positions, pessaries, etc., many of which will be recognized, some of which are new.

W. L. D.

ARTICLE XIX.—THE DESCRIPTIVE ATLAS OF ANATOMY. A Representation of the Anatomy of the Human Body; in 92 Royal quarto plates containing 550 figures; pp. vii-92, 11. Philadelphia: J. B. Lippincott & Co. London: Smith, Elder & Co. 1880.

This atlas is anonymous, a poor recommendation in medicine. "Every figure has been carefully revised by a Metropolitan Hospital surgeon and a successful teacher of Anatomy in one of the chief London medical schools." He should have drawn the figures himself, we are sorry to say, for some are ill-executed: the vulva, for instance, looks as if it had been drawn from imagination, not from nature.

It is a cheap book, with too darkly shaded cuts, indistinct types, and many abbreviations not supplemented in the page. It does not come up to the standard of the plates of Gray's Anatomy, and must prove of little value to students in this country, while practitioners need better works. It is a blunder to paint bones black, and use black types over them. It is useless to precede the names of every muscle by an *M* when the cut represents nothing else.

"Hepar, stomachus, cystis fellea, cavum peritonei," etc., are obsolete, as well as the lobus dexter and lobus sinister of the liver. The coloring of arteries and veins is an old idea devoid of advantages, since the color is so little in the living subject. The only advantage of that atlas is cheapness and a multiplicity of figures. H. D. V.

ARTICLE XX.—A PRACTICAL TREATISE ON SURGICAL DIAGNOSIS, DESIGNED AS A MANUAL FOR PRACTITIONERS AND STUDENTS. By Ambrose L. Ranney, M.D., Adjunct Professor of Anatomy, and Lecturer on Minor Surgery in the Medical Department University of New York. Wm. Wood & Co., 1879; 386 pp.

As is implied in the title, this book touches upon points of Differential Diagnosis only, omitting all questions of Ætiology, Pathology and Treatment.

By reading across the page the contrasting points are well brought out, while the particular symptoms are shown from above downward. The reader will be well repaid for his study of fractures, dislocation, hernia and tumors.

Dr. Ranney has arranged a valuable book for the profession.

J. H. T.

ARTICLE XXI.—THE CAUSES AND RESULTS OF PULMONARY HÆMORRHAGE, WITH REMARKS ON TREATMENT. By Reginald E. Thompson, M.D. Illustrated; 130 pp. London: Smith, Elder & Co., 1879.

Upon the subject of this volume the author has expended, as he tells us, much diligence and many years of study and investigation. It is the outcome of the physical examination of over twenty-two thousand patients while living, and over three hundred of the same patients *post mortem.* The opening chapters treat in a condensed way of the anatomy of the lung, the pathogeny of hæmorrhage, while the body of the book is made up of clinical cases illustrating inherited predisposition to bleeding, causes, physical signs, sequelæ, consequences, treatment, etc.

It is an interesting, readable monograph upon the subject of Pulmonary Hæmorrhage, and should be read by all. J. H. T.

ARTICLE XXII.—DISEASES OF THE THROAT AND NOSE, INCLUDING PHARNX, LARYNX, TRACHEA, ŒSOPHAGUS, NASAL CAVITIES, AND NECK. By Morell Mackensie, M.D., London; Senior Physician to the Hospital for Diseases of the Throat and Chest; Lecturer on Diseases of the Throat, at the London Hospital Medical College, etc. Vol. I. Diseases of the Pharynx, Larynx, and Trachea. 8vo, pp. 570, cloth. Philadelphia: Presley Blakiston. 1880.

Dr. Mackensie's book is as good as, and not any better than, many works of the same kind. A good classification of subjects, useful references to authors who treated them before, a vast experience at the command of the author, the exclusion of superfluous matter, are so many advantages which will make of it a reference book of much usefulness. But it is one of those works which each old lecturer must produce on his special subject once in his life. It may prove very useful to the House Physician to the "Hospital for Diseases of the Throat and Chest," may be to physicians in general, in England; but it will not answer the purpose of the average American practitioner, on account of its deficiency in what relates to treatment.

There is a superfluity of etiology and diagnosis in our days with a lamentable scantiness of the methodical treatment of each disease in its various forms as met with in this country, and this is precisely what the profession in America is craving for, and what the book of Dr. Mackensie lacks; although it would be a good addition to a large library. It is well printed, has very few typographical errors for a first edition, and enough pretty cuts to illustrate the subject. H. D. V.

ARTICLE XXIII.—DIPHTHERIA: ITS CAUSES, PREVENTION AND PROPER TREATMENT. By J. H. Kellogg, M.D. Good Health Publishing Co., Battle Creek, Mich.; 64 pp.

This small work is one of a series of popular medical literature, and aims to so educate the public mind on the matter of early treatment of the disease as to render efficient aid to the physician when called. Certainly there is great need of a good deal of popular knowledge on this subject, for the fragmentary articles published in the family papers go but a small way towards educating the people in this matter.

In so small a work, in our opinion, it is hardly necessary to describe and arrange the disease into classes—all phases of but the same disease.

It is, on the whole, a book needed in the household, of service to those having children, and should be widely circulated.

J. H. T.

ARTICLE XXIV.—A GUIDE TO THE PRACTICAL EXAMINATION OF URINE, FOR THE USE OF PHYSICIAN AND STUDENT. By James Tyson, M.D., Professor of General Pathology and Morbid Anatomy in the University of Pennsylvania. Third Edition, revised and corrected. With illustrations. Philadelphia: Lindsay & Blakiston, 1880; 183 pp.

Dr. Tyson has in this edition simply rearranged somewhat his previous editions, making a very neat and compact manual on the subject of Urinalysis.

Some of the less important matter of the two previous editions has been omitted, with the view of keeping the size of the volume within its original limits.

The cuts are good, some of the older and poorer ones being replaced by some more satisfactory. The book is so bound as to lie open upon the laboratory table ready for reference. It can be recommended as reliable and convenient. J. H. T.

ARTICLE XXV.—HANDBOOK OF SYSTEMATIC URINARY ANALYSIS—CHEMICAL AND MICROSCOPICAL. By Frank M. Deems, M.D., Laboratory Instructor in Medical Department, University of New York. New York: Industrial Publishing Co., 1880; 30 pp.

Will afford valuable aid to both student and practitioner. Can be recommended to all. J. H. T.

ARTICLE XXVI.—THE MICROSCOPIST'S ANNUAL FOR 1879. Edited by John Phin. New York: The Industrial Publishing Co., 1880; 48 pp.

This is a handy little volume to have; contains complete list of microscopical societies, dealers in apparatus and accessories, postal information compiled especially for microscopists, duties on imported instruments, etc. To be issued annually.

